# Why Do Woodpeckers Resist Head Impact Injury: A Biomechanical Investigation

**DOI:** 10.1371/journal.pone.0026490

**Published:** 2011-10-26

**Authors:** Lizhen Wang, Jason Tak-Man Cheung, Fang Pu, Deyu Li, Ming Zhang, Yubo Fan

**Affiliations:** 1 Key Laboratory for Biomechanics and Mechanobiology of Ministry of Education, School of Biological Science and Medical Engineering, Beihang University, Beijing, People's Republic of China; 2 Department of Health Technology and Informatics, the Hong Kong Polytechnic University, Hong Kong; 3 Li Ning Sports Science Research Center, Beijing, People's Republic of China; University of Plymouth, United Kingdom

## Abstract

Head injury is a leading cause of morbidity and death in both industrialized and developing countries. It is estimated that brain injuries account for 15% of the burden of fatalities and disabilities, and represent the leading cause of death in young adults. Brain injury may be caused by an impact or a sudden change in the linear and/or angular velocity of the head. However, the woodpecker does not experience any head injury at the high speed of 6–7 m/s with a deceleration of 1000 g when it drums a tree trunk. It is still not known how woodpeckers protect their brain from impact injury. In order to investigate this, two synchronous high-speed video systems were used to observe the pecking process, and the force sensor was used to measure the peck force. The mechanical properties and macro/micro morphological structure in woodpecker's head were investigated using a mechanical testing system and micro-CT scanning. Finite element (FE) models of the woodpecker's head were established to study the dynamic intracranial responses. The result showed that macro/micro morphology of cranial bone and beak can be recognized as a major contributor to non-impact-injuries. This biomechanical analysis makes it possible to visualize events during woodpecker pecking and may inspire new approaches to prevention and treatment of human head injury.

## Introduction

Head injuries remain as an increasingly common cause of death and severe disabilities around the world [1]–[3]. Considering the competitive team sports at the 2004 Olympic Games, it was shown that 24% of all the injuries reported were head injuries [4]. According to European Brain Injury Consortium (EBIC) survey, 51% of head injuries were from car-crash accident or sports related to fall [5], [6]. Yet an intriguing example from nature is the case of woodpeckers *(Picoides)*, who drum tree trunks at a speed of 6–7 m/s with a deceleration of approximately 1000 g, but no head injuries [7]–[9]. Indeed, woodpecker drums about 10–20 bouts continuously, and every bout takes about 50 milliseconds. It drums about 12,000 times per day on average. Woodpeckers perform rhythmic drumming with their beaks on surfaces such as dead tree limbs to catch and feed themselves with worms, or attract a mate and announce their territorial boundaries [9]. In view of biomechanics it is not well understood why woodpeckers resist head impact injuries.

Several research groups have studied the mechanism of resist impact injuries in woodpecker's head [7]–[15]. Earlier classic ornithological studies suggested two principal factors. The unique anatomical structures of woodpecker's head have been thought as one of factors. The unique anatomical structures included stout sharply pointed beaks [10]; A long tongue called hyoid bone which originates from the dorsum of the maxilla, passes through the right nostril, divides into two parts between the eyes, and the dividends then arch over the superior portion of the skull and around the occiput by passing on either side of the neck, coming forward through the lower mandible, and uniting into one again below the forehead [7], [9], [11]; Narrow subdural space and little cerebrospinal fluid (CSF), relatively small and smooth brain specially oriented to allow larger contact areas within the skull [7], [12], [13]. Meanwhile, the straight-line pecking trajectory in the sagittal plane was suggested to be against rotational forces as the protective mechanism that rotational, rather than translational, accelerations produce concussion [8], [14], [15]. However, little attention has been paid to the three-dimensional (3D) kinematic/kinetic features and quantitative estimation of macro/micro morphology and histology on woodpecker's head such as beak and cranial bone. There is overwhelming evidence that bone mass and micro-architecture are sensitive to the mechanical stimuli, such that make its mechanical behavior both in microstructure and strength adapt to the environmental changes [16]–[20]. Here, we investigated 3D kinematics, mechanical properties, macro/micro morphological structure and dynamic response of woodpecker's head quantitatively. The purpose of this study was to investigate the role of 3D kinematics, macro/micro structures of beak and cranial bone in avoiding impact injury of woodpecker's head.

## Materials and Methods

This study was approved by the Science and Ethics Committee of School of Biological Science and Medical Engineering in Beihang University, China (Approval ID: 20090301). Great Spotted woodpecker *(Dendrocopos major)* was selected for its wide distribution in the northern China. For comparison, Eurasian hoopoe *(Upupa epops)*, a related bird with comparable size that pecks on insects inside the soil mainly, was also selected to be compared with woodpecker. They were fed with yellow mealworm *(Tenebrio molitor L.)* in separate metal cages.

**Figure 1:**
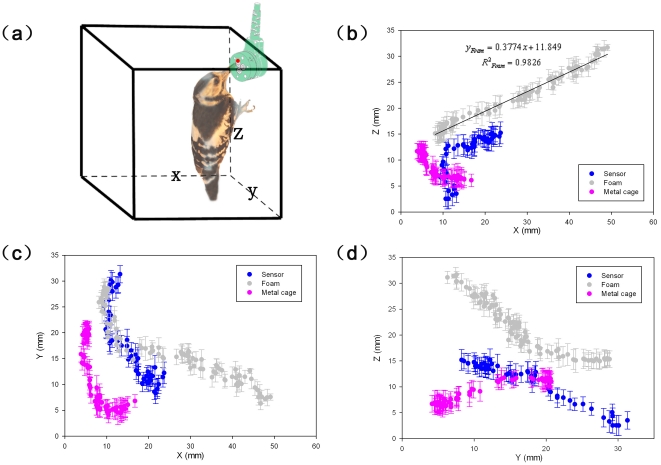
The 3D pecking trajectory during woodpecker's pecking. (a) 3D schematic diagram; (b) Pecking trajectory on the sagittal plane; (c) Pecking trajectory on the transverse plane; (d) Pecking trajectory on the coronal plane.

To investigate the pecking behavior, the 3D motion of Great Spotted Woodpeckers and Eurasian hoopoe during pecking were captured using two synchronous high-speed cameras of 2,000 frames per second (fps) (Photron Fastcam SA-3, USA). The resolution was set to 512×512 pixels. Meanwhile, pecking force was collected synchronously using a force/torque sensor (ATI Force/Torque Sensor: nano17, USA). The sensor, foam and metal cage were set as the peckable objects. The selected points on the typical anatomical location such as abdomen, eyelid and tip of beak were traced (Fig. 1a). Kinematic parameters such as moving trajectory, the time of a typical cyclic pecking process, pre-impact velocity, and the deceleration of the two kinds of birds were obtained.

Then, quantitative analysis of micro-morphology of the cranial bone was done based on image processing of micro-computed tomography (micro-CT, Skyscan1076, Skyscan, Belgium) and scanning electronic microscopy (SEM, JSM-6490, JEOL, Tokyo, Japan) respectively. The micro-structural parameters such as bone volume fraction (BV/TV), structure model index (SMI), trabecular thickness (Tb.Th), trabecular number (Tb.N), trabecular separation (Tb.Sp), bone mineral density (BMD), defined in Table 1, were calculated from micro-CT images. Data analysis was conducted by means of SPSS 16 software. The spatial resolution for specimen scanning was set to 18 µ*m*. Meanwhile, the micro-structures of specimens were washed with normal saline to remove blood, mucus or tissue fluid, and dehydrated in an up-grading series of ethanol concentration from 30% to 100%, finally sputter-coated with an approximately 20 nm-thick layer of gold. Then, the micro-structures were examined *in vitro* using SEM.

**Table 1:** Definitions of various micro-structural parameters analyzed in this study.

	Parameters Abbrev	Definition (Units)
Bone volume fraction	BV/TV	Relative percentage of bone within 3-D ROI (%)
Structural model index	SMI	Quantification of relative shape of trabeculae from rod-like to plate-like
Trabecular thickness	Tb.Th	Quantification of relative thickness of individual trabeculae within 3-D ROI (µm.)
Trabecular number	Tb.N	Quantification of relative number of individual trabeculae within 3-D ROI (1/mm)
Trabecular separation	Tb.Sp	Quantification of relative spacing between individual trabeculae within 3-D ROI (µm)
Bone mineral density	BMD	3-D derivation of mineral density (g/cm^3^)

**Figure 2:**
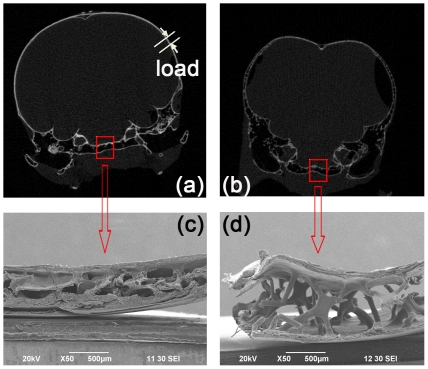
Micro-morphology of cranial bone. (a) The micro-CT scanning images of Great Spotted woodpecker's head on the coronal plane; (b) The micro-CT scanning images of Eurasian hoopoe's head on the coronal plane; (c) The SEM image of Great Spotted woodpecker's cranial bone; (d) The SEM image of Eurasian hoopoe's cranial bone.

To study the mechanical properties of woodpecker's cranial bone and beak, destructive compressive mechanical tests were carried out on 12 specimens (4×4×0.4 mm^3^) with a material testing machine (MTS 858, MTS Systems Corporation, USA) using the 50N and 1 kN load cells respectively. The specimens were placed between two steel loading rods with low friction using low-viscous mineral oil as a lubricant. The direction of compressive load was shown in Fig. 2a. After being pre-conditioned, the specimen was compressed at a constant strain rate of 0.2% s^−1^, until a compression of 3% was reached [21]. From the stress-strain curve, the ultimate strength and the Young's modulus were calculated as the tangent of the stress-strain curve at a strain of 0.6% [22].

To investigate the dynamic response of woodpecker's head, a geometrically accurate 3D FE model of woodpecker's head, including the upper/lower beak, skull, brain and hyoid bone, was developed based on the actual geometry and anatomic detail from micro-CT scanning. The material properties of skull, beak and hyoid bone were derived from the data in above mentioned mechanical test. A homogenous density and linearly viscoelastic material model in combination with a large-deformation theory was chosen to model the brain tissue [23]–[25]. The behavior of this material was characterized as viscoelastic in shear with a deviatoric stress rate dependent on the shear relaxation modulus, while the compressive behavior of the brain was considered as elastic. The shear characteristic of viscoelastic behavior of brain was expressed by:
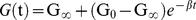
(1)

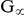
 is the long term shear modulus, G_0_ is the short term shear modulus and is a decay factor.

The numerical simulation was performed with the dynamic FE commercial package LS-Dyna version 971 (Livermore Software Inc.) [26]. The FE predicted results were compared with the corresponding test results during impact in order to validate the FE model. The whole head was collided with a rigid wall at an initial velocity of 1 m/s with the duration of 10–20 milliseconds (ms) based on the kinematics recording. The quantitatively studies have been done by analyzing the time histories of effective stress on the skull, brain and the tip of upper/lower beak under the initial velocity; the effects of hyoid bone and the length of beak on the dynamic response at the selected points of brain using the FE method. The same model was used to simulate the beak_lower_>beak_upper_, beak_lower_ = beak_upper_ and beak_lower_<beak_upper_ by changing the length of beak. All other conditions are the same as those used in the three simulations.

## Results

As shown in Table 2, both linear and angular accelerations occurred during pecking. The peak linear velocity and deceleration of woodpecker were significantly higher than those of Eurasian hoopoe. The peak angular velocity and deceleration of woodpecker were closer to that of Eurasian hoopoe. The kinematic parameters were different when woodpecker struck to varied objects. Fig. 1 (b,c,d) reveals the 3D trajectory of the tip of woodpecker beak when it pecked different objects. When woodpecker pecked on foam, it moved along straight line on the sagittal plane during impact. However, when it pecked sensor and metal cage, curved trajectory was observed.

**Table 2:** The peak linear and angular impact velocities, decelerations of Great Spotted woodpecker and Eurasian hoopoe.

	Pecking object	Peak linear velocity (m/s)	Peak linear deceleration (m/s^2^)	Peak angular velocity (rad/s)	Peak angular deceleration (Krad/s^2^)
Great Spotted Woodpecker	foam	7.572	9790	160	297
	metal cage	2.736	4171	336	448
Eurasian Hoopoe	foam	2.460	3612	158	296
Pileated Woodpecker^7^	tree trunk	7.490	13680	-----	-----

As shown in Fig. 2 (a, b), silhouette of woodpecker skull on the coronal plane was smooth and close to ellipsoid compared to Eurasian hoopoe; The brain of woodpecker was tightly packed by relatively dense cranial bone comprising of cortical and spongy bone with less spongy bone compared to Eurasian hoopoe. Fig. 2 (c,d) shows the SEM images of spongy bone on the occipital of the two birds. More plate-like spongy bones were observed in woodpecker's cranial bone, while more rod-like for Eurasian hoopoe. The micro-structural parameters were presented as means±standard deviation (SD) based on the micro-CT images in Table 3. The differences of these micro-structural parameters except Tb.Sp were found to be statistically significant (*p* = 0.05) between Great Spotted woodpecker and Eurasian hoopoe.

**Table 3:** Micro-structural parameters of the occiput of Great Spotted Woodpecker and Eurasian Hoopoe.

	Great Spotted Woodpecker(means ± SD)	Eurasian Hoopoe (means ± SD)	p-value
Bone volume fraction [ BV/TV (%)]	8.587±1.673	4.562±0.799*	0.023
Structural model index [SMI]	1.194±0.311	1.561±0.225*	0.035
Trabecular thickness [Tb.Th(µm)]	190±18	127±15*	0.041
Trabecular number [Tb.N(1/mm)]	0.506±0.123	0.411±0.086	0.067
Trabecular separation [Tb.Sp(µm)]	451±286	712±213*	0.017
Bone mineral density [BMD(g/cm^3^)]	0.218±0.015	0.101±0.011*	0.012

*p<0.05.

**Figure 3:**
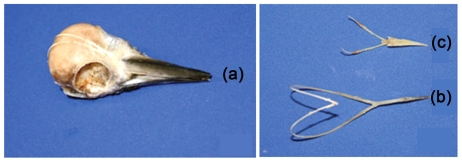
Anatomical structures of head and hyoid bone. (a) Great Spotted woodpecker's head; (b) Great Spotted Woodpecker's hyoid bone; (c) Eurasian hoopoe's hyoid bone.

**Figure 4:**
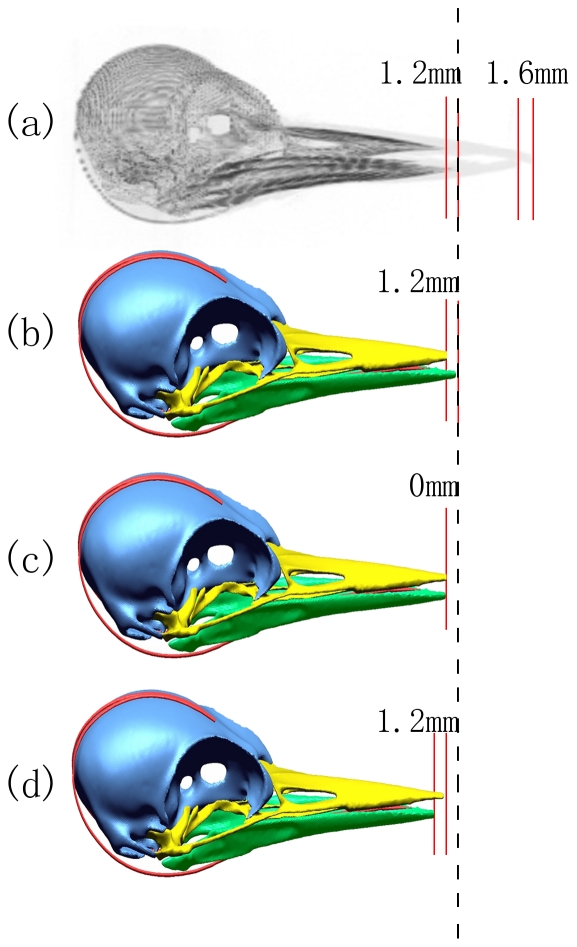
Micro-CT image and the FE models of Great Spotted Woodpecker' head. (a) Micro-CT image of Great Spotted Woodpecker' head; (b) Beak_Lower_>Beak_Upper_ FE model; (c) Beak_Lower_ = Beak_Upper_ FE model; (d) Beak_Lower_<Beak_Upper_ FE model.

As shown in Fig. 3a,b, the hyoid bone, a sling-like structure only in woodpecker grows all the way up to the top of the head and into the nasal cavity where the sheath fuses to nasal membrane, which was up to about 80 mm; and it was longer than the tongue of Eurasian hoopoe (Fig. 3c). Also, the outer tissue layer of upper beak was 1.6 mm longer than that of the lower beak; on the contrary, the bone structure of the upper beak was about 1.2 mm shorter than the lower beak (Fig. 4a). The FE model of woodpecker's head with varied length of upper/lower beak were developed, as shown in Fig. 4b,c,d.

**Figure 5:**
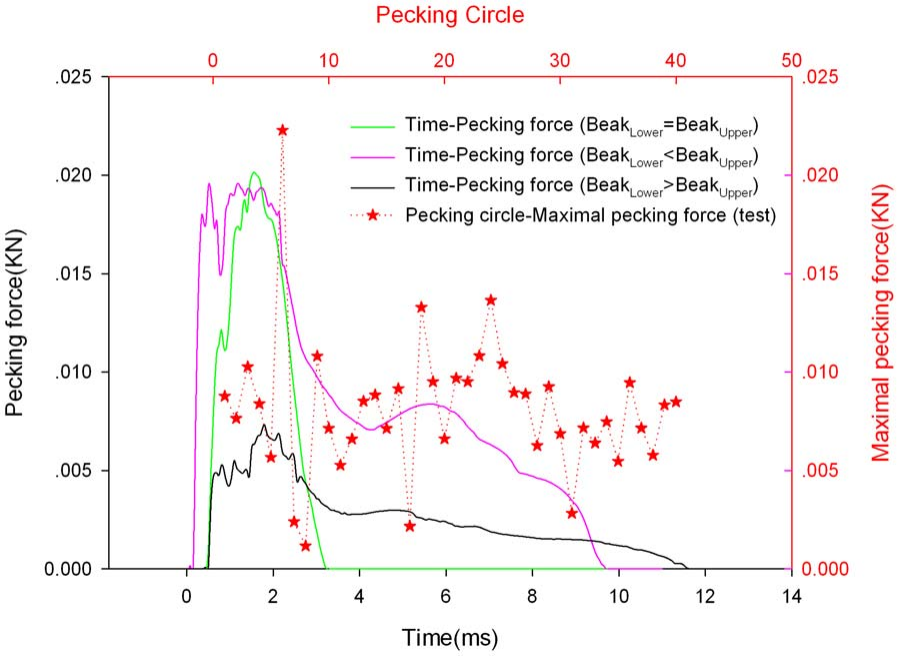
The pecking force-time histories at the initial velocity of 1 m/s for both the FE analysis and pecking circle-maximal pecking force in the experiment.

As listed in Table 4, the material properties of skull and beak, hyoid were derived from the data in above mentioned mechanical test. The pecking force-time histories at initial velocity of 1 m/s are shown in Fig. 5 for both the FE analysis and test. Forty pecking circles were selected randomly from many reproductive bouts in the experiments. The pecking force was about 8.1±3.5N in the experiment. The maximal pecking force was 7.3N in the beak_Lower_>beak_Upper_ FE model. Good correlation was obtained in the predicted responses of the FE model compared with the corresponding experimental results during impact.

**Table 4:** The material properties of woodpecker's skull, beak, hyoid bone and brain.

	Skull	Beak	Hyoid bone	Brain
Young's modulus(GPa)	0.31	1.00	1.13	---
Coefficient of Poisson	0.4	0.3	0.2	---
Density ρ(kg/m3)	---	---		1040
Bulk modulus K (Gpa)	---	--		0.5
Short time shear modulus G_0_ (GPa)	---	---		5.28E-04
Long time shear modulus G∞(GPa)	---	---		1.68E-04
Time constant β(s^−1^)	---	---		35

**Figure 6:**
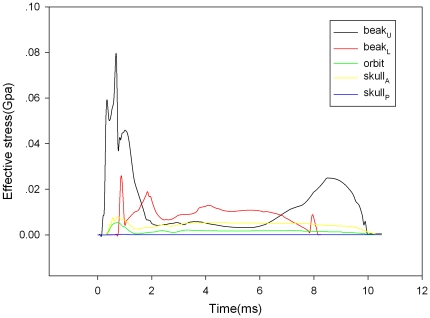
The time histories of effective stress at the selected points on beak, orbit and skull. (Beak_U_-the point on the tip of upper beak; Beak_L_-the point on the tip of lower beak; Orbit-the point on the orbit; Skull_A_-the point on the anterior part of skull; Skull_P_-the point on the posterior part of skull).

**Figure 7:**
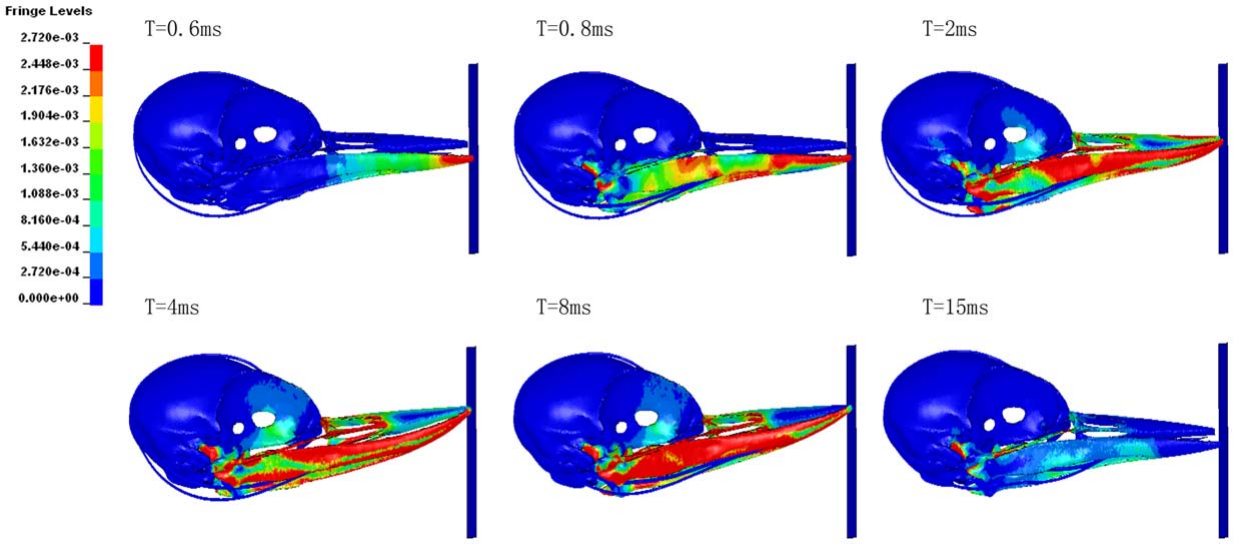
The effective stress distribution of woodpecker's head during pecking.

**Figure 8:**
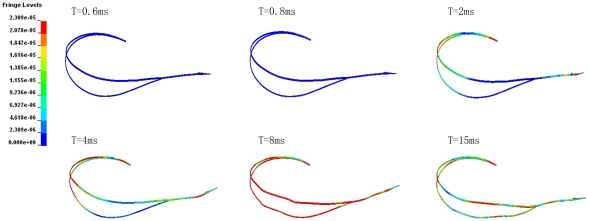
The effective stress distribution of the hyoid bone during pecking.

Fig. 6 shows that the time histories of the effective stresses at the selected points. The maximum stress on upper/lower beak was about 2–8 times of orbit or skull. Table 5 shows that consistently higher strains induced especially at the frontal part of brain when the length of lower beak is equal to the upper beak during impact. As shown in Fig. 7 and Fig. 8, the stress distribution of woodpecker's head and hyoid bone in the process of impact. The stress concentration has been observed obviously in the orbit.

**Table 5:** The predicted peak strain at the selected points on brain during pecking.

Peak strain ( 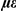 )	Three location on the brain		
	Point1	Point2	Point3
BeakLower>BeakUpper	0.04	0.02	0.06
BeakLower = BeakUpper	0.69	0.03	0.11
BeakLower<BeakUpper	0.08	0.05	0.18

Point1:anterior-brain; Piont2:posterior-brain; Point3:inferior-brain.

## Discussion

Woodpeckers peck with the high speed and deceleration [7]–[9]. We presumed that woodpeckers were protected against acceleration-deceleration-impact-related head injury, although no studies have been carried out to prove it comparatively. Simple reasoning would indicate that if the woodpeckers got headaches, they would stop pecking.

To clarify the reason that woodpeckers have no head injury, we investigated 3D kinematics, mechanical properties, macro/micro morphological structure and dynamic response of woodpecker's head quantitatively in view of biomechanics. Our findings showed that woodpeckers possess protective mechanisms for its self-adjusted behavior and the special anatomical structure. It moved along a linear trajectory on the sagittal plane at the moment of collision when woodpecker pecked on foam, which was consistent with the previous studies [8], [9], [27]. However, it was observed that the rotational components generated obviously on the coronal and horizontal planes. The peak angular velocity and deceleration of woodpecker were closer to those of Eurasian hoopoe. In light of the above observations, both of the Great spotted woodpecker and Eurasian hoopoe could resist rotational force in general. Hence, the centripetal theory of the straight trajectory in preventing brain injury in woodpeckers was doubtful. It was also founded that the peak linear velocity and deceleration for woodpecker were higher than those of Eurasian hoopoe significantly. The kinematic parameters were different when woodpecker struck to other objects such as sensor and metal cage. The results indicated that woodpecker has better performance of self-adjusted consciously to resist linear impact-related injury. Then, there should be some other characteristics to protect the brain from impact injuries caused by linear and rotational force.

Established reasonable correlations between bone elastic modulus, strength and structural parameters derived from micro-CT images showed that the elastic properties of cranial bone could be estimated by measuring its volume fraction (or density) [28]–[31]. According to the images of micro-CT, more plate-like spongy bones were observed on woodpecker skull, while more rod-like on Eurasian hoopoe. And the distribution of spongy bone was uneven in woodpecker's skull. It was rich in the forehead and occiput, not in other parts. The special anatomical structures included the long hyoid bone and the unequal length of upper/lower beak on the outer tissue layer and the inner layer separately. Above mentioned features in woodpecker's head may be contributed to bearing high stress or absorbing shock stress resulted from pecking or probing.

Evidence shows that sudden changes of relevant mechanical parameters in terms of effective stress, shear strain and stress, and relative motion between brain and skull do indeed cause surface contusions, concussion, diffuse axonal injury (DAI) as well as acute subdural hematoma [32]–[35]. Shear deformation of the brain due to head rotation has long been postulated as a major cause of brain injury since brain tissue has the low shear stiffness [14], [36]. Unfortunately, the measurement of stress or strain was almost impossible during an impact, particularly *in vivo*. Alternatively, FE method can be adopted. Previous studies had developed FE model of woodpecker's head based on 2D measurement of head, and relevant mechanical parameters of human [7], [9], [37], [38]. The model in this study has the exact 3D geometry obtained from micro-CT images, and the measured elastic modulus of woodpecker's skull and beak may make the results closer to the biological reality.

The pecking force-time histories at initial velocity of 1 m/s are shown in Fig. 5 for both the FE analysis and experimental test. The correlation of predicted responses obtained in the FE model and experiment during impact was good. There were two peaks in the simulation results. The first peak was due to the longer beak touch, while the second peak occurred due to shorter beak impact on the rigid wall. It was found that the minimal impact force occurred under the condition of beak_lower_>beak_upper_ during impact.

Two points at frontal and occipital on the anterior and posterior of skull, two points on the tip of upper/lower beak and one point on the orbit were selected respectively to study the time history of the effective stress. The time histories of the effective stresses at all of the selected points are shown in Fig. 6. Interestingly, maximum effective stress and shear stress concentration of woodpecker's skull always occurred on orbit at the moment of collision (Fig. 7, T = 4 ms), which would associated with the observation of eyelid shut before impact, then opened immediately captured by high-speed videos. In addition, the occurrence time of maximum stress was later than that of beak and skull. It did not work until the end of collision (Fig. 8). It seems that the hyoid bone may play a role of safety belt to woodpecker's head to some extent.

Parametric analysis was done by changing the relative length of the upper and lower beak in the developed FE model (Fig. 4b,c,d) to evaluate the biomechanical effects during pecking. It was expected that the length variation of upper and lower beaks would influence the impact mechanics and load transmission. Brain injury was shown to correlate with strain and strain rate [39]. Shock strains at the frontal and occipital of brain were analyzed utilizing the present models. By comparing the FE predicted strain on the anterior and posterior of brain, as well as the inferior of brain during impact (Table 5), it was found that upper and lower beaks with equal lengths consistently induced higher strains at all of the three locations on woodpecker's brain.

In this study, a finite element model of woodpecker's head was developed to understand the effect of different factors on the load transfer in the process of pecking. The 3D model was first applied to simulate the process of pecking, and validated by experimental tests. The pecking force predicted by the model was in good agreement with the experimental observation and test data. It provided a solid platform for parametric analysis. The effects of various factors were evaluated in order to draw a conclusion on how woodpeckers resist from impact injury. The conclusions of the present study are summarized as follows.

The special macro/micro morphological structures in woodpecker's head including the hyoid bone, the uneven plate-like spongy bones and unequal length of upper/lower beak were major factors to non-impact-injuries. The long hyoid bone has played a role of safety belt to woodpecker's head especially after impact. The outer tissue layer covering the upper beak was 1.6 mm longer than that of the lower beak; on the contrary, the high-strength bone structure of the upper beak was about 1.2 mm shorter than the lower beak. Beak morphology was found to affect impact force, brain strain. It was shown that most of the pecking forces were always carried by the longer beak during pecking.

As described above, woodpecker's sophisticated shock absorption system is a good cooperative phenomenon, not any single factor being able to achieve the function. The design of intelligent helmet or impact-related injury resistant device would be enlightened greatly by the optimizations of woodpecker's skull morphology and microstructure and is helpful in developing new concepts for minimizing head impact injuries in future work.
